# Vector induced skewing of antibody Fc-effector functions

**DOI:** 10.1186/1742-4690-9-S2-P361

**Published:** 2012-09-13

**Authors:** A Chung, A Dugast, H Robinson, Y Chan, ME Ackerman, J Cox, W Koff, D Barouch, S Rerks-Ngarm, N Michael, J Kim, G Alter

**Affiliations:** 1 Ragon Institute of MGH, MIT & Harvard, Boston, MA, USA; 2 Thayer School of Engineering, Dartmouth,, Hanover, NH, USA; 3 International AIDS Vaccine Initiative, New York, NY, USA; 4 Beth Israel Deaconess Medical Center, Boston, MA, USA; 5 Department of Disease Control, Ministry of Public Health, Nonthaburi, Thailand; 6 US Military HIV Research Program, Silver Spring, MD, USA

## Background

The RV144 vaccine showed a moderate efficacy of protection from HIV infection. The major immune response induced by RV144 was non-neutralizing HIV-specific antibodies (Abs), that may have potentially mediated Ab Dependent Cellular Cytotoxicity (ADCC) and/or Ab dependent Cellular Phagocytosis (ADCP). However little is known about the potential role of different vaccine regimens on inducing these types of humoral immune responses, and whether particular vaccine approaches may preferentially induce robust innate immune recruiting antibody activity that could confer more protection against infection. We therefore aimed to characterize the antibody-effector functional profiles of antibodies elicited by a number of different vaccine approaches including those induced in the: VAX003 trial (bivalent rgp120 clade B/E), RV144 (ALVAC vCP1521 + rgp120 B/E), IPCAVD001 (rAd26.ENVA.01), IAVI-C002 (MVA), IAVI-P002 (DNA + MVA) and IAVI-V001 (DNA + rAd5).

## Methods

Abs were purified from the plasma or serum of vaccinees. IgGs were then assayed for ADCC, ADCP, NK degranulation and cytokine production, antibody isotype selection, and Ab affinity for Fc-receptors ( FcγRIIa, FcγRIIb and FcγRIIIa).

## Results

IAVI-C002 and IAVI-P002 vaccination induced negligible Fc-mediated innate immune responses, while IAVI-V001 was able to induce ADCP in 33% of vaccines. IPCAVD001 was also able to induce strong ADCP in 90% of subjects, but only weak ADCC, NK degranulation or cytokine release. Interestingly, only RV144 and VAX003 vaccination induced strong ADCC, ADCP, NK degranulation and cytokine responses. Furthermore, Abs induced by RV144 and IPCAVD exhibited a more polyfunctional profile compared to VAX003, associated with a skewed isotype distribution of HIV-specific Abs and selective Fc-receptor affinity binding profile.

## Conclusion

These data suggest for the first time that distinct vaccine regimens, and vaccine vectors, may selectively induce antibodies with Fc-enhanced functional profiles able to elicit polyfunctional antibody responses, that may provide improved protection from infection.

